# Global transcriptional responses of *Pseudomonas syringae *DC3000 to changes in iron bioavailability *in vitro*

**DOI:** 10.1186/1471-2180-8-209

**Published:** 2008-12-02

**Authors:** Philip A Bronstein, Melanie J Filiatrault, Christopher R Myers, Michael Rutzke, David J Schneider, Samuel W Cartinhour

**Affiliations:** 1 United States Department of Agriculture-Agricultural Research Service, Robert W Holley Center for Agriculture and Health, Ithaca, NY 14853, USA; 2 Department of Plant Pathology and Plant-Microbe Biology, Cornell University, Ithaca, NY 14853, USA; 3 Computational Biology Service Unit, Life Sciences Core Laboratories Center, Cornell University, Ithaca, NY 14852, USA; 4 Department of Food Science, Cornell University, Ithaca, NY 14853, USA

## Abstract

**Background:**

*Pseudomonas syringae *pv *tomato *DC3000 (DC3000) is a Gram-negative model plant pathogen that is found in a wide variety of environments. To survive in these diverse conditions it must sense and respond to various environmental cues. One micronutrient required for most forms of life is iron. Bioavailable iron has been shown to be an important global regulator for many bacteria where it not only regulates a wide variety of genes involved in general cell physiology but also virulence determinants. In this study we used microarrays to study differential gene regulation in DC3000 in response to changes in levels of cell-associated iron.

**Results:**

DC3000 cultures were grown under highly controlled conditions and analyzed after the addition of iron citrate or sodium citrate to the media. In the cultures supplemented with iron, we found that cell-associated iron increased rapidly while culture densities were not significantly different over 4 hours when compared to cultures with sodium citrate added. Microarray analysis of samples taken from before and after the addition of either sodium citrate or iron citrate identified 386 differentially regulated genes with high statistical confidence. Differentially regulated genes were clustered based on expression patterns observed between comparison of samples taken at different time points and with different supplements. This analysis grouped genes associated with the same regulatory motifs and/or had similar putative or known function.

**Conclusion:**

This study shows iron is rapidly taken up from the medium by iron-depleted DC3000 cultures and that bioavailable iron is a global cue for the expression of iron transport, storage, and known virulence factors in DC3000. Furthermore approximately 34% of the differentially regulated genes are associated with one of four regulatory motifs for Fur, PvdS, HrpL, or RpoD.

## Background

*Pseudomonas syringae *pv *tomato *DC3000 (DC3000), a pathogen of tomato and Arabidopsis, is a Gram-negative bacterium that is found in a wide variety of environmental niches. This bacterium must to be able to interpret environmental cues to determine if it is in the soil, in water drops, on a plant surface, or in the plant apoplast. The pathogen adapts to each local environment by altering its internal physiology, including changing mRNA, protein content, and internal metabolite stores. By altering its behavior DC3000 is better able to scavenge available nutrients, withstand various environmental stresses and, if necessary, coordinate expression and deployment of appropriate virulence factors. For growth within plants DC3000 can express two major virulence factors: the phytotoxin coronatine, which causes chlorosis in plants [1], and a type III secretion system (T3SS), whose main function is to help the bacteria to evade host defenses by interfering with intracellular signaling pathways of plant cells (for recent review [2]). Several regulatory proteins have been identified that control T3SS and coronatine production but the precise nature of the chemical and physical factors used as environmental cues involved in the expression of these pathways are unknown.

One environmental signal detected by many bacterial pathogens, including *Pseudomonas aeruginosa*, is iron. Iron homeostasis in *P. aeruginosa *has been investigated due to its essential role in opportunistic human disease, especially the expression of extracellular virulence determinants including an exotoxin and proteases [3,4]. Many iron-responsive regulatory pathways have been studied in this species. One key regulator is the ferric uptake regulator, Fur [5,6]. Fur forms a homodimer complex in the presence of Zn^2+ ^and Fe^2+ ^ions [7-9] enabling it to bind to specific DNA motifs, "Fur boxes". These motifs are overlapping inverted repeat sequences that often occur in close proximity to, or overlap with, promoters. Fur binds to DNA in iron-replete conditions leading to repression of downstream transcriptional units. The characteristic repressor behavior of Fur is clearly evident in microarray studies of patterns of expression of iron-responsive genes in *P. aeruginosa *PAO1 (PAO1) [10,11]. Recently the Fur regulon has been studied using computational methods to identify putative Fur regulated genes that specifically contain cooperative upstream binding sites [12].

The regulatory networks that respond to iron are complex. For instance in PAO1 there are several extracytoplasmic function (ECF) sigma factors and small RNAs (sRNAs) that are associated with iron homeostasis [11,13,14]. Twelve sigma factor genes have confirmed or putative upstream Fur binding sites, and nine of these genes exhibit iron-responsive expression under one or more conditions [10,11]. Sigma factors modulate transcription by recruiting core RNA polymerase to promoters, which in turn regulate many genes. A recent study in PAO1 characterized several ECF sigma factors that control TonB-dependent receptors and a variety of putative metal transport systems [14].

Previous studies in PAO1 have implicated more than 300 genes that are differentially expressed in response in iron [10,11]. Many of the regulatory elements and downstream targets found in PAO1 have homologs in other pseudomonads. However, relatively little direct experimentation has been reported for *P. syringae *pathovars regarding iron homeostasis. The iron-dependent production of toxins in *P. syringae *pv *syringae *has been known for some time [15,16]. Loper and Lindow, using *P. syringae *31-derived strains showed limited iron bioavailability in colonized regions of leaf surfaces [17]. In addition, Joyner and Lindow found considerable variation in iron bioavailability on plant leaves [18]. Analysis of the DC3000 genome sequence revealed the existence of two extracellular siderophores, yersiniabactin and pyoverdin, along with multiple putative TonB-dependent siderophore receptors, putative iron uptake pathways, and proteins involved in iron storage [19]. Recent studies show that both yersiniabactin and pyoverdin are produced in DC3000 culture and that yersiniabactin is also produced in planta [20]. Our group has also recently characterized the PvdS regulon, which is the regulon of an ECF type sigma factor that controls genes responsible for the production of pyoverdin along with other iron responsive genes [21]. These studies imply that iron acquisition is important for growth in planta and that iron may be an important environmental signal sensed by DC3000. However, since a strain lacking in the production of yersiniabactin has no growth defect in the plant and an increased growth rate in low-iron media, the role of environmental iron signaling and acquisition is likely to be a complex phenomenon [20].

In this study we evaluated the effect of bioavailable iron on bacterial gene transcription using carefully controlled growth conditions to maximize differential cell associated iron levels and reduce confounding environmental and growth effects. We measured cell-associated iron concentrations in DC3000 at multiple time points after the addition of iron citrate or sodium citrate and evaluated the transcriptome to identify differentially regulated genes. RNA levels from each sample were analyzed using microarrays. We found that cell-associated iron levels are strongly associated with the differential expression of a variety of pathways in DC3000 including siderophore production, iron transport systems, sigma factors, T3SS and coronatine production. The differentially expressed genes were grouped by their patterns of regulation and we found that genes that clustered together had similar known or putative functions and shared bioinformatically derived regulatory motifs. Based on these results, we believe that many previously uncharacterized genes can be assigned putative functions and we present an initial systems view of iron-dependent regulation in DC3000.

## Results

### Kinetics of iron uptake by iron-depleted *P. syringae *cultures

**Figure 1:**
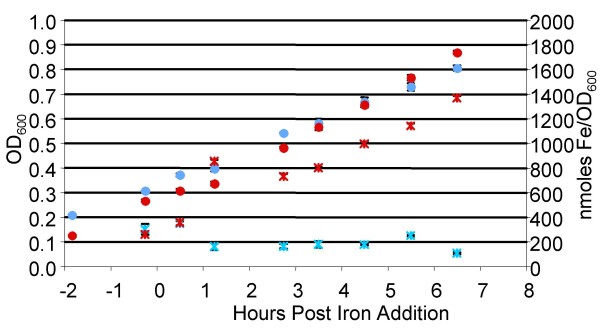
**Effects of iron on cultures grown in minimal media**. DC3000 cultures were grown in MG medium. Culture density was measured by spectrophotometry after the addition of 20 μmoles of ferric citrate or sodium citrate for an increase of 50 μM ferric citrate (red circles) or sodium citrate (blue circles) over the background iron levels in the media (~17 nmoles). Cell associated iron was measured from these cultures by ICP-ES (red x: ferric citrate added; blue x: sodium citrate added).

In order to optimize growth and reduce the amount of variation introduced by using conventional flask culture, a bioreactor system was used to grow DC3000 in a minimal medium, mannitol-glutamate (MG) [22]. MG supports moderate expression of both major virulence factors in DC3000, type III secretion and coronatine biosynthesis (data not shown). To produce a bacterial culture in an iron-depleted state we harvested DC3000 grown on modified Luria broth (LM) plates to inoculate bioreactors with a low level of inoculum (final OD_600 _= 0.01). Inductively Coupled Plasma Emission Spectroscopy (ICP-ES) was used to determine the total iron content in the culturing medium and cell-associated iron levels. By using high purity components we were able to obtain a medium with 42.9 ± 18.0 nM of iron. After cultures were grown overnight to an OD_600 _~0.3 the cell-associated iron levels were found to be 0.278 ± 0.003 μmole/OD_600 _of iron, which we defined as an iron-limited condition. Cultures were then supplemented with ferric citrate to raise the final iron concentration by 50 μM, or an equimolar amount of sodium citrate. While rapid uptake of iron can be seen in those cultures that received iron citrate (Figure 1), the OD_600 _measurements between the cultures do not greatly differ out to four hours. We therefore used the four-hour time point in subsequent experiments to maximize the dynamic range of cell-associated iron levels while minimizing growth differences between cultures supplemented with either sodium citrate or iron citrate.

### Reproducibility of culture growth and iron uptake

**Figure 2:**
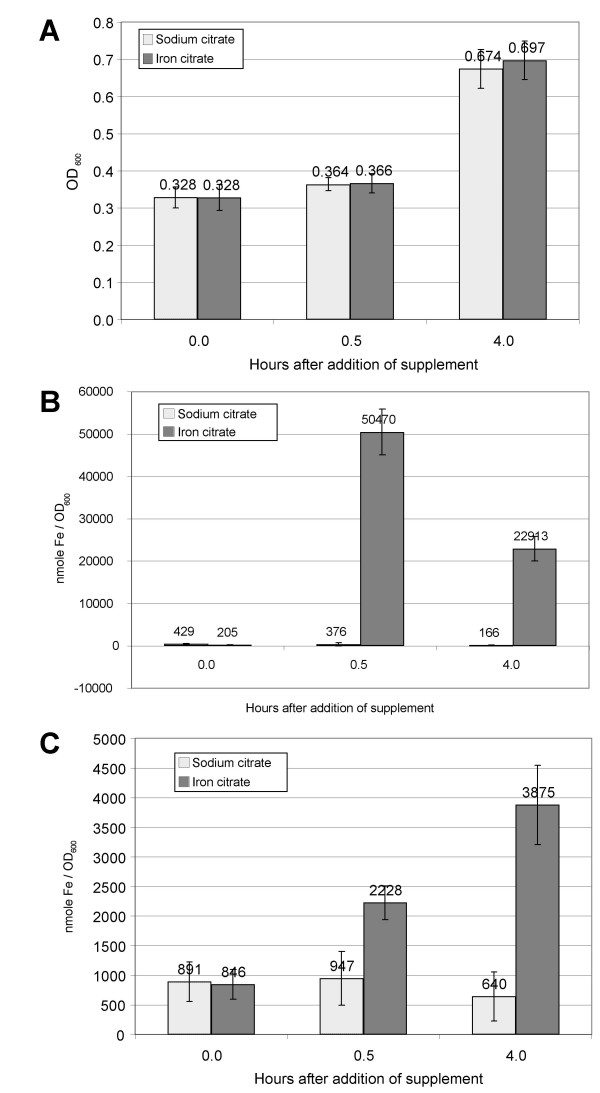
**Culture characteristics of DC3000 grown in bioreactors**. DC3000 cultures were grown in MG media in bioreactors to an OD_600 _of ~0.3 then either ferric citrate or sodium citrate was added to increase the final concentrations by 50 μM. Samples were taken from these reactors before addition of citrate compounds and 30 minutes and 4 hours after their addition. Culture densities were measured by spectrophotometry (A). Cell associated (B) and extracellular (C) iron levels were measured by ICP-ES. Error bars represent variations across 5 biological replicates.

To evaluate transcriptional responses to iron in DC3000, five replicate cultures were grown as described above and samples were taken immediately before the addition of iron or sodium citrate, then 30 minutes and 4 hours after the addition. As expected, culture densities did not significantly differ between the two cultures at the same time points (Figure 2A) while the cell-associated iron differed greatly at these time points (Figure 2B). High levels of iron were still present at 4 hours in culture supernatants that had been supplemented with iron citrate, demonstrating these cultures remained iron replete throughout the course of the experiment (Figure 2C). RNA was isolated from all samples and was used for all subsequent experiments in this study. The tight reproducibility of these cultures allowed us to use a very stringent statistical analysis of the microarray data presented below.

### Global expression changes determined via microarray analysis

**Figure 3:**
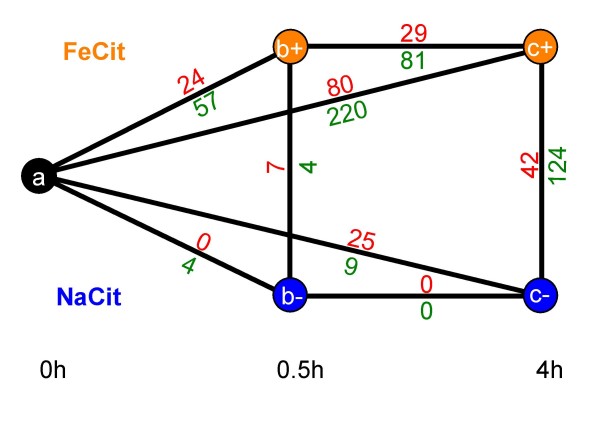
**Numbers of differentially expressed transcripts in pair-wise comparisons**. Cultures were grown in bioreactors in MG medium. Samples were taken at three time points: before (a) the addition of either sodium citrate(-) or ferric citrate(+), 30 minutes (b) and 4 hours (c) after the addition of supplements. RNA from these samples were processed and hybridized to Affymetrix-style microarray chips from NimbleGen. Statistically significant differential expression was defined using GCRMA/FDR analysis with p = 0.01. The number of up-regulated (red) and down-regulated (green) transcripts between condition 1 (node to left or above) and condition 2 (node to right or below) with respect to orientation of the numbers are shown.

To evaluate the transcriptome of DC3000 in response to environmental iron, mRNA from three time points and two conditions for five biological replicates was analyzed using Affymetrix-style arrays from NimbleGen. The microarrays contained oligomer probes for 5608 genes as well as many intergenic (IG) regions on chromosome. The RNA isolated from the samples described above was converted to cDNA and hybridized to the chips as described in Materials and Methods. QC analysis of the microarrays was performed using previously defined methods [23] (data not shown). Pair-wise comparisons between different time points and culture conditions were made and a GCRMA/FDR analysis with a stringency of p = 0.01 was chosen to evaluate differentially expressed genes (Figure 3). For these analyses we removed probe sets of genes that were found multiple times in the genome, such as transposases, and IS elements. We also removed IG sequences that did not contain i) predicted genes, described after the initial DC3000 annotation [19], or ii) sRNAs predicted by Rfam [24]. At a p value equal to or less than 0.01, 386 genes were differentially expressed in one or more comparisons. The fold change of these genes ranged from approximately 1.1-fold to 2850-fold. 376 genes showed differential regulation, in one or more comparisons, of 1.2-fold or greater and 315 genes showed differential expression of greater than 1.5-fold. In fact, even with a highly restrictive p value (less than or equal to 0.0004) 140 genes were still identified as differentially expressed. The 386 differentially expressed genes identified include 16 putative or confirmed regulatory genes, 67 hypothetical genes, and 196 genes with proposed functions or putative functional motifs. A complete list of differentially expressed genes and their fold changes at different p values can be found in Additional File 1.

**Figure 4:**
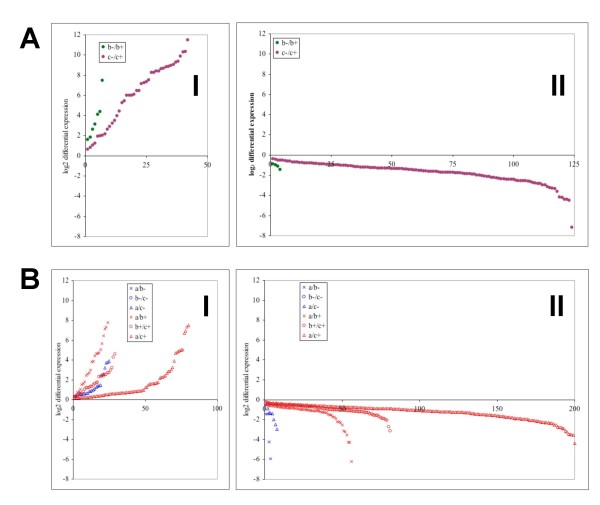
**Scatter plot of transcripts sorted by mean differential expression level**. Differentially expressed transcripts are arranged from the most repressed to the most induced in each comparison. The y-axis is the log_2 _differential expression as defined previously (GCRMA/FDR p = 0.01) and the x-axis indicates the number of the up (I) or down (II) regulated gene arranged by magnitude of change. Comparisons shown are between samples at the same time point with different supplements (A) or between samples at different time points but have received the same supplement (B).

Our experimental design made it possible to consider each comparison individually and characterize each set of differentially expressed genes separately (Figure 3). For example, analyses can focus on sets of genes that are reacting to specific stimuli such as genes that are differentially expressed 30 minutes after the addition of iron, or genes that are affected by growth from OD_600 _~0.3 to OD_600 _~0.7. The fold change of the genes in each comparison can be seen in Figure 4A and 4B. Not surprisingly the greatest number of differentially expressed genes, and the greatest magnitude of changes, occurred between the comparisons of 4 hours with and without the addition of iron (Figure 4A) and between time 0 and 4 hours after the addition of iron citrate (Figure 4B). Most genes repressed by iron in the comparison between cultures 4 hours after the addition of sodium citrate or iron citrate are involved in siderophore production, iron transport and storage. Genes that are induced by iron in this same comparison include genes involved in T3SS, coronatine synthesis, virulence regulators *hrpRS, hrpL*, and *corR*, and others whose products use iron as a co-factor. The differential regulation of several genes at the 4 hour time points, including *hrpRS*, *hrpL*, *corR*, and *pvdS*, was confirmed using qRT-PCR (data not shown). Many genes that were differentially expressed between cultures 4 hours after the addition of sodium citrate or iron citrate were also found in the comparison of the 0 hour and 4 hour time points after the addition of iron citrate. However other genes are unique to the 0 hour to 4 hour time point comparisons. These include *rpoS, rpoE, sigW*, and 7 genes that encode for putative regulatory proteins.

### Clustering of differentially expressed genes

**Figure 5:**
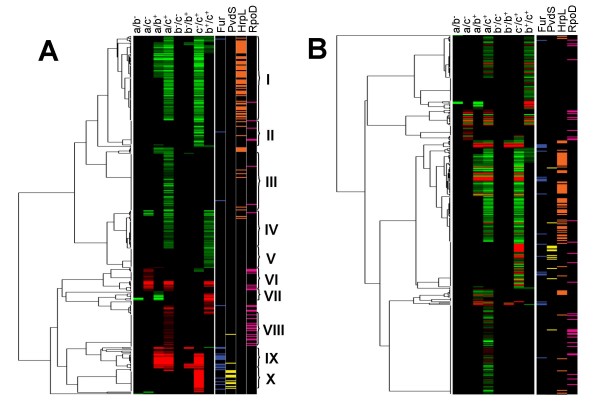
**Clustering of transcripts based on patterns of differential expression**. Differentially expressed transcripts were clustered using pair-wise average linkage with un-centered correlation distance (A) or pair-wise average linkage with absolute un-centered correlation distance (B). Each column of the left represents a comparison as described in Figure 3. Red bars represent up-regulated transcripts and green bars represent down-regulated transcripts. Intensity of color is directly related to magnitude of differential expression. The presence of a computationally derived regulatory motif upstream of the transcript containing the probes for the differentially expressed gene is denoted on the right for the regulators Fur (blue), PvdS (yellow), HrpL (orange), and RpoD (purple). Roman numerals represent transcript clusters highlighted in the discussion section.

To fully exploit the data generated, we considered how each individual gene behaves in all tested conditions and grouped genes that react similarly. Because multiple time points from samples grown under two conditions were evaluated, differentially expressed genes could be clustered based on similar expression patterns. We clustered the data using pair-wise average linkage with un-centered correlation distance, which allows for the grouping of genes with the same relative expression change. This should group genes with similar function and/or regulation with their putative positive regulators (Figure 5A). Additionally we clustered genes with pair-wise average linkage with absolute un-centered correlation distance, which should cluster similarly expressed genes with those that could represent negative regulators (Figure 5B). See also Additional Files 2 through 5 to view clustering analysis using Java TreeView or similar product.

### Evaluating gene clusters with regulatory motifs

After clustering we correlated the results with putative regulatory motifs generated by bioinformatics from other available data sets. Four motifs were used to search the DC3000 genome: the HrpL associated motif from Ferreira, *et al*., the PvdS motif from Swingle, *et al*., a Fur binding motif derived from PAO1 microarrays, and a RpoD motif generated by our group from unpublished proteomics data from DC3000 [10,11,21,25]. Genes were considered associated with the HrpL or PvdS motifs if they were reported as such in the previous publications [21,25]. For the RpoD and Fur motifs, genes were considered associated with these motifs if i) the motif was within 220 base pairs of the putative start of the first gene in the transcript (operon) containing the gene, as defined by a curated set of operons derived from VIMSS Operon Predictions [26], and ii) in the case of RpoD, if the motif and the putative transcript were in the same orientation (see Additional File 6 for RpoD and Fur motifs). Our analyses predicted 98 HrpL associated, 70 PvdS associated, 90 Fur associated, and 448 RpoD associated genes in the DC3000 genome.

Approximately 34% of the differentially expressed genes (140 of 386) were associated with one of these four motifs, and many genes associated with a particular regulatory motif grouped closely in the microarray-derived clusters. These include 16 differentially expressed operons (21 genes) that were associated with the Fur motif, 8 operons (13 genes) with the PvdS motif, 46 operons (74 genes) with HrpL, and 21 operons (32 genes) with the putative RpoD motif. As expected, Fur motifs were mostly associated with genes that showed differential expression at early time point comparisons after the addition of iron (8 out of 16 operons in the b-/b+ or a/b+ comparisons), while genes associated with the PvdS motif were associated with differential expression at later time points in response to iron (12 genes were differentially expressed in the c-/c+ comparison while none were identified in the b-/b+ comparison and only 1 was identified in the a/b+ comparison). Most of the HrpL associated genes are more highly expressed in iron supplemented media (a/c+ or c-/c+ comparisons). There appears to be a subgroup of HrpL associated genes that are also responsive to iron at earlier time points. The early set is diverse and includes some genes that encode for the Hrp secretion apparatus and Hrp secreted proteins. Additionally, the early set does not completely overlap with genes that were induced early in previous HrpL regulatory studies [25]. Finally the RpoD motif is generally associated with genes that have lower expression at later time points (higher culture density). Many of these (27 of 33) have less than 2-fold differential expression, including genes that encode for ribosomal proteins, all of which show less than 1.2 fold change.

**Figure 6:**
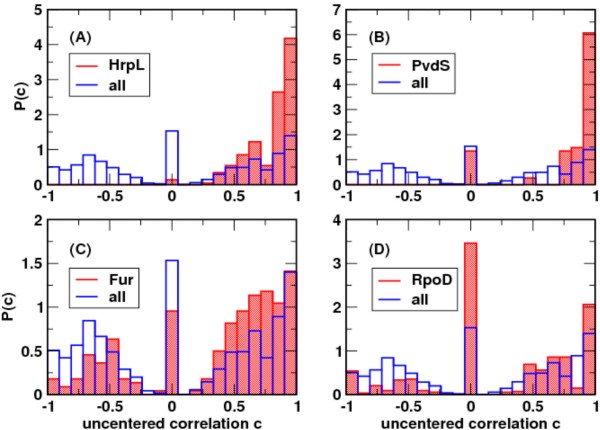
**Correlation of expression patterns and presence of regulatory motifs**. Histograms representing the distribution of pairwise expression correlations for all pairs of genes within a co-regulated set, as compared to the distribution among all pairs of differentially expressed genes (regardless of putative regulation). The expression correlation c(i, j) between two genes i and j is the uncentered correlation metric used for clustering of gene expression in Figure 5A (i.e., the cosine of the angle between the expression vectors for genes i and j). Distributions are (red) over all pairs of genes within a specified set, or (blue) over all 386 genes in the full differentially expressed set. (A) HrpL; (B) PvdS; (C) Fur; (D) RpoD. The sizable spike at c = 0 (i.e., representing uncorrelated gene pairs) in the background distribution results from the sparse nature of the data array: for a gene that is not deemed to exhibit significant differential expression in a given condition, its fold change is set to zero for the purposes of clustering and computing expression correlations.

A quantitative summary of the correlation of differential expression patterns and the presence or absence of predicted regulatory motifs is shown in Figure 6. For each of the four regulators considered, the distribution of pair-wise expression correlations within the predicted co-regulated set was compared to the distribution of correlations between all pairs of differentially expressed genes. For all four regulatory motifs, genes predicted to be regulated by the same factor have more highly correlated expression than in the full set of genes. By sampling from the background distribution of all pair-wise gene correlations (see Materials and Methods for details), we can estimate, for each factor, a p-value that reflects the chance the observed expression correlations could have been drawn at random from the background distribution. These p-values are: HrpL, p < 10^-5^; PvdS, p < 0.00023; Fur, p < 0.023; RpoD, p < 0.016. The sets of genes predicted to be regulated by HrpL and PvdS are both more highly correlated in their expression and less consistent with the background distribution. This might arise from both intrinsically tighter regulation by these factors and the fact that those models have been derived by direct experimental evidence. In contrast the Fur and RpoD models are less well validated and the correlations are not as striking. Overall, the p values for all motifs are less than 0.025 and demonstrate that the clustering analysis has successfully grouped genes with putative regulatory motifs closely together.

## Discussion

**Figure 7:**
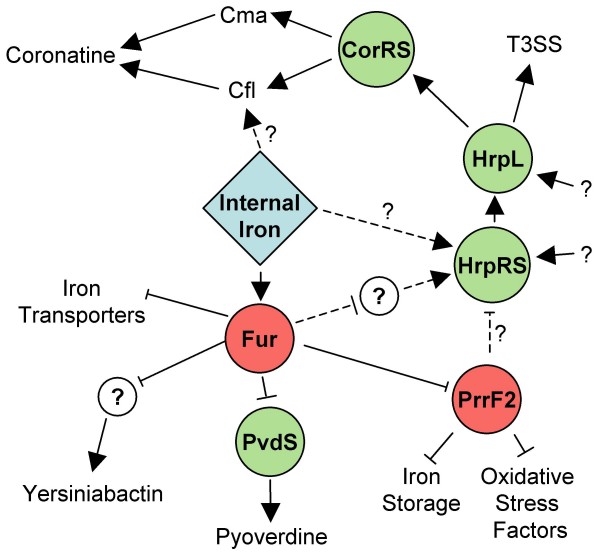
**Simplified model of proposed features in iron regulation**. This model shows proposed and known regulatory actions in DC3000 in response to internal iron concentrations. Red circles represent negative regulators and green circles denote positive regulators. Dotted lines represent proposed regulatory actions while solid lines indicate actions reported in the literature. Question marks indicate unknown regulators, mechanisms or environmental signals that may be involved in global iron responses.

In this study we examined the association between bioavailable iron and gene transcription in the bacterial plant pathogen *Pseudomonas syringae *pv *tomato *DC3000. We developed a bioreactor-based protocol to obtain highly reproducible growth of DC3000 in a medium that allowed for the expression of known virulence factors. Conditions were selected to maximize cell-associated iron concentrations and minimize confounding effects of changes in growth states. By analyzing samples from different time points in two different conditions we were able to cluster genes based on their patterns of differential expression such that genes with similar proposed function and regulatory motifs were closely associated. The remainder of the discussion will highlight genes in several clusters of differentially expressed genes and the known or suspected pathways present within these clusters, both of which provide clues concerning how the bacterium is altering its physiology to respond to bioavailable iron. An overview model of proposed key regulatory events in response to iron is shown in Figure 7.

### HrpL regulon cluster

Cluster I from Figure 5A contains a total of 91 differentially expressed genes that are induced in high iron conditions, 61 of which are associated with an HrpL regulatory motif. Other genes encode for bacterioferritins, superoxide dismutase, catalases, and peroxidase. There are also 6 hypothetical genes in this cluster and 4 known regulators: *hrpRS, hrpL*, and *corR *[27-29]. Additionally there are 3 putative response regulators and 1 putative sensor histidine kinase that may be involved in expression of virulence factors, iron storage or coping with free oxygen radicals.

While it is reported that iron influences T3SS in *Shigella *and *Salmonella *[30,31], this is the first report of bioavailable iron regulating T3SS in the plant pathogen DC3000. The mechanism by which iron modulates T3SS seems to differ between *Shigella *and *Salmonella*. In *Shigella *a Fur regulated small RNA, RyhB, is implicated in regulating T3SS transcription [30]; while in *Salmonella *Fur, through an unknown mechanism, affects the activity of HilD, an AraC-like regulator, while in turn regulates expression of T3SS [31]. A *ryhB *homolog in DC3000, *prrF2*, is associated with a Fur motif and was differentially regulated in response to iron in our experiments and experiments in *P. aerginosa *[13]. If PrrF2 acts as a negative regulator of T3SS, which has been reported for RyhB [32], then we would expect *prrF2 *to co-cluster with T3SS regulatory genes in analyses using pair-wise average linkage with absolute un-centered correlation distance (Figure 5B). In this analysis we found that *prrF2 *clustered closely with T3SS genes including *hrpL *and *hrpS*, both known regulators of T3SS in DC3000. However, further experiments are required to characterize the role of *prrF2 *in the regulation of T3SS genes.

### Coronatine production clusters

Cluster II contains 29 genes that are up-regulated in response to iron after 4 hours. Some encode known or putative enzymes that contain iron as a co-factor. These include genes involved in the production of succinate semialdehyde, a precursor for coronatine [33]. While there are no putative regulatory proteins found in this cluster, 9 genes are described as hypothetical.

The 61 genes in cluster III are differentially expressed in the comparison between cultures supplemented with iron citrate at the 0 and 4 hour time points. Several T3SS substrates appear in this cluster as well as 4 genes involved in the formation of coronafacic acid, a precursor to coronatine, and one (*gabD-3*) that is involved in formation of succinate semialdehyde [33]. Additionally there are genes encoding for porins, heat shock proteins, and many putative ABC transporters. About a third of the genes in this cluster are differentially expressed less than 1.5 fold. There are 15 hypothetical genes in the cluster, 3 of which have homology to functional domains of transcriptional regulators.

### Growth phase dependent clusters

Four distinct clusters appear to be regulated in a growth phase dependent manner. The first of these, cluster IV, is up-regulated between 0 hour and 4 hour time points and/or 30 minute and 4 hour time points in the presence of iron citrate. Of the 41 genes in this cluster, 8 are differentially expressed less than 1.5 fold in both comparisons. These include several involved in general metabolism. Additionally there are two putative regulators in this set; one encodes for a PrtN-like regulator found only in *Pseudomonas syringae *pathovars (PSPTO_0570) and the other encodes for a protein homologous to OmpR. PrtN regulates pyocins in *Pseudomonas aeruginosa *[34,35] and PSPTO_0570 is found within a region contains a putative F-type pyocin and at least one other gene, PSPTO_0569, clusters closely in PSPTO_0570 in this analysis. There are also two known regulators, OmpR, and RpoS. Since RpoS is reported to up-regulate genes at the onset of stationary phase [36,37], we hypothesize that these regulators control the expression of many of the genes found in this cluster. However, we have not been able to derive regulatory motifs from the upstream regions of the genes in this cluster or other known RpoS regulated genes in other pseudomonads.

Cluster V contains 20 genes and 1 regulator, *glnK*, which controls expression of genes involved in nitrogen metabolism. Cluster V also includes two genes thought to be involved in nitrogen metabolism and several genes predicted to be involved in mannitol or amino acid transport [38,39]. We hypothesize that GlnK is responsible for the differential regulation for many of these genes.

The next cluster (VI) contains 25 genes that are repressed at later time points. Seven show less than a 1.5 fold change and eight are associated with the RpoD motif. Many of these are involved in general metabolism. One interesting gene, PSPTO_4442, is homologous to *bolA *in *E*.*coli*. The BolA protein is hypothesized to be involved in the switch between cell elongation and septation and is induced during the transition into early stationary phase [37].

The last growth phase dependent gene cluster (VIII) contains 35 genes that are repressed between 0 hours and 4 hours after the addition of iron citrate. Almost half (15) show less than a 1.5 change between these two conditions and nine of those exhibit 1.2 fold or less change. Many are involved in ribosomal synthesis. This suggests that the cultures were beginning to transition from exponential growth and early stationary phase at this time point. 14 of the genes in this cluster are associated with the predicted RpoD motif. Because our RpoD motif contains a weak -35 box, a strong -10 box and a conserved motif directly downstream of the -10 box, this cluster may represent a subset of RpoD regulated genes that are subjected to additional regulation by other factors. Two other regulatory genes in this cluster, *algU *(*rpoE*) and *sigX*, are involved in outer membrane modifications and exhibit less than a 1.5 fold change in this comparison [40,41].

### Citrate uptake cluster

This cluster (VII) of 8 genes includes 3 hypothetical genes and is up-regulated starting at 30 minutes in response to the addition of either iron citrate or sodium citrate. Four genes are homologous to genes involved in citrate uptake (*tctABC*, and a citrate permease, *citM*) [42,43]. While we cannot identify any regulatory proteins among these genes, we speculate that the 3 hypothetical genes that cluster in this group are also involved in citrate uptake.

### Iron uptake and storage cluster

Cluster IX contains genes that are down-regulated in response to the addition of iron citrate. Thirteen of the twenty-three genes in this cluster are associated with a Fur motif. These include three genes that encode for putative lipoproteins, two hypothetical genes, and other genes whose homologs have been implicated as iron-responsive in other bacteria. Cluster IX also includes two regions in the genome predicted to contain small RNAs, both of which were included in the microarray analysis. One sRNA designated here as *cobalamin-4*, was identified by the Rfam database as a cobalamin riboswitch [24]. The cobalamin riboswitch terminates transcription of downstream genes if B12 is bound to the riboswitch [44]. In DC3000, *cobalamin-4 *is upstream of an operon containing PSPTO_3256-PSPTO_3258 that is not differentially regulated in our analyses, even at p = 0.05. The genes are predicted to encode for members of an iron ABC transport system [19] but without further analysis it is impossible to rule out that these genes are involved in the transport of cobalamin. Therefore, we hypothesize that the promoter upstream of *cobalamin-4 *is differentially expressed in response to iron but since cobalamin is still present in the cell in sufficient amounts (data not shown), B12 is binding to the RNA and attenuating the transcription of the downstream genes.

The other sRNA found in this cluster is *prrF2*. As stated previously *prrF2 *is a homolog of *ryhB *and has been characterized in *P. aeruginosa *for its role in iron homeostasis [13,45]. In Figure 5B* prrF2 *clusters with genes that are similar to genes in *P. aeruginosa *that are affected by *prrF1/2 *including genes involved in catalase production, bacterioferritins, and superoxide dismutase [45]. This leads us to believe that, in addition to possibly regulating T3SS, *prrF1/2 *play similar roles for iron homeostasis in DC3000 as they do in *P. aeruginosa*.

Also included within cluster IX are genes that encode for the TonB transport system (*exbB-1, exbD-1*,*tonB-1*) and putative TonB-dependent siderophore receptors (PSPTO_2152, 3294, 3574). Because of the co-clustering with the TonB transport system and the strong association with the Fur motif, we propose that the putative lipoproteins and hypothetical proteins encoded by genes in this cluster are involved in iron-uptake processes.

### Siderophore production cluster

Cluster X contains 25 genes that are repressed in response to iron after 4 hours. Twenty of these genes are known or hypothesized to be associated with either yersiniabactin (8 genes) or pyoverdine (12 genes) production or siderophore transport/uptake [19,20,46]. Additionally the regulator for pyoverdine production, *pvdS*, is found within this cluster. There are also 2 genes that encode for putative TonB-dependent siderophore receptors (PSPTO_2605, 3462). PSPTO_2605 is located on the chromosome next to the yersiniabactin synthesis genes and is probably responsible for the uptake of that siderophore. Finally, *fecB*, involved in iron dicitrate transport, is also found within this group[47,48]. We hypothesize that genes within this cluster are involved in iron acquisition and that intracellular iron levels at these time points are close to "iron-saturated". If so, we expect genes involved in iron uptake to be down-regulated by regulators downstream from Fur regulation, such sigma factors like PvdS.

## Conclusion

In order to be a successful pathogen, a bacterium must sense and respond to a diverse array of environmental signals. Many signals cause the differential regulation of hundreds of genes by primary and downstream regulatory events. In this study we have investigated the connection between multiple bacterial regulons and iron availability using a systems biology approach by integrating global expression analysis with computational biology. By analyzing samples taken from cultures with different cell associated iron concentrations at multiple time points, we have attempted to unravel complex regulatory pathways. We found that clustering differentially expressed genes based on their patterns of expression grouped of genes with like function together. We also used regulatory motifs derived from other data sets to show that many closely grouped genes also share common regulatory features. This global study has allowed us to hypothesize on functions for many previously uncharacterized genes base on clustering and has given us an initial systems level view of gene regulation in response to bioavailable iron of *Pseudomonas syringae *pv *tomato *DC3000.

## Methods

### Media preparation and Bacterial growth in bioreactors

A Sixfors bioreactor system (Infors, Sweden) was used for culturing bacteria for microarray experiments. Reactors were soaked overnight in 20% nitric acid to removed residual bound iron. The 500 ml reactors were thoroughly washed and 400 ml of defined minimal medium [Mannitol-Glutamate (MG) media (10 g/L of mannitol, 2 g/L of L-glutamic acid, 0.5 g/L of KH_2_PO_4_, 0.2 g/L of NaCl, 0.2 g/L of MgSO_4_, final pH of 7)] was added [22]. When available, Sigma Ultrapure components were used to minimize the amount of iron contamination in the media. Reactors were autoclaved and allowed to oxygenate for at least 4 hours prior to inoculation. Running conditions were as follows: 25°C, 1 L/min of air supplied via sparging, and a Rushton impeller spinning at 500 RPM for additional perturbation of the media. Vessels were inoculated to an OD_600 _of 0.01 with bacteria that had been grown to confluency on LM agar [49] and then resuspended in MG medium prior to inoculation.

### Sample collection and Isolation of RNA

When cultures reached an OD_600 _of 0.3 (~16 hours after inoculation), samples were taken (t = 0 h) and iron citrate (Sigma, St Louis, MO) or sodium citrate (Sigma) was added to a final concentration of 50 μM. At each time point (t = 0 h, t = 0.5 h, and t = 4 h) 35 ml of culture was taken from each reactor via an aseptic method. Twenty-five ml of the cultures was centrifuged to separate bacteria from the supernatant and each fraction was frozen at -20°C for further analysis of iron levels. Five ml of culture was pelleted by centrifugation at room temperature for 5 min at 10,000 × *g *and the supernatant was removed. RNA was isolated using the RNeasy kit (Qiagen, Carlsbad, CA) following manufacture's instructions; with the exception that lyzozyme was used at a concentration of 5 mg/ml. RNA was treated with DNase I (Ambion, Austin, TX) to remove residual DNA and then cleaned and concentrated using the MinElute kit (Qiagen). Removal of DNA was verified by qRT-PCR [50]. Integrity of the RNA was assessed using the Agilent Bioanalyzer (Microarray Core Facility, Cornell University).

### Measurement of iron concentrations

Bacterial pellets from 25 ml of culture were digested with 1.0 ml of concentrated nitric acid at 120°C until dry, then 1.0 ml of a 1:1 mixture of concentrated nitric acid and perchloric acid was added and heated at 220°C until dry. The ash was dissolved in 20.0 ml of 5% Nitric acid and analyzed on an axially viewed ICP trace analyzer emission spectrometer (model ICAP 61E trace analyzer, Thermo Electron, Waltham Ma). The transfer optics were replaced with a short depth of field transfer optics to reduce matrix effects, (2000) US Patent No. 6,122050. A specialized spray chamber and desolvation system (2005) US Patent No. 6859272 was also used.

### Microarray processing

The microarrays used in this study were NimbleExpress Made-to-Order Prokaryotic Arrays for *P. syringae *pv *tomato *DC3000 (part# 530131) (NimbleGen Systems Inc., Madison, WI, USA) purchased through Affymetrix (Santa Clara, CA, USA). These microarrays consisted of 190,000 probes, with probe length of 24 nucleotides, representing 5,608 genes, as well as several intergenic regions, with a minimum of 17 probes/gene, including perfect match and mismatch probes. The NimbleExpress arrays are packaged in an Affymetrix GeneChip cartridge (49 format), and can be used with GeneChip reagents and processed on the GeneChip Instrument System. cDNA synthesis, labeling, and fragmentation were performed at the Microarray Core Facility at Cornell University using standard Affymetrix protocols. Hybridization, staining, washing, and chip scanning were also performed at the Microarray Core Facility using the Flexmidi_Euk2v3 fluidics protocol from Affymetrix with a modification in wash temperature B on a Fluidic station FS450. Microarray chips were scanned on a GeneChip scanner 3000-7G with 4-color upgrade and array autoloader.

### Analysis of Microarray data

Microarray data were analyzed using Bioconductor [51]. Quality assessment of microarray data was performed as previously described [23]. Redundant and unwanted probes were removed from data sets prior to final statistical analysis. Samples were taken from 5 biological replicates for all time points. All data was normalized using gcrma and pair wise comparisons were made using genefilter requiring at least 3 chips where probe sets had intensity above 10. Subsequent FDR analysis was done using multtest at different p values (0.5, 0.1, 0.02, 0.004). Differentially expressed genes were clustered using the Bio.Cluster module in BioPython (version 1.44). The resulting dendograms were visualized using Java TreeView software [52].

### Regulatory motifs

Our group previously published HrpL and PvdS motifs [21,25]. The microarray data on iron-responsive genes in PAO1 from Ochsner et al. [11] and Palma et al. [10] was used to identify putative Fur binding motifs in all pseudomonads. The process of identifying putative Fur binding sites is a variant of computational methods used previously by our group [25,50,53]. Briefly, training sets were constructed from the manually annotated set from Ochsner et al. and by performing Gibbs sampling with various constraints on motif length and symmetry on regions upstream of iron-responsive genes in the microarray experiments of Ochsner et al. and Palma et al. The HMM was calibrated and used to scan each available pseudomonad genome sequence. The RpoD promoter model and putative regulatory targets were derived from unpublished proteomics data. From the set of proteins that were present in a preliminary proteomics survey, a list of candidate genes and upstream regulatory elements were assembled, based on operon predictions used and reported in [25]. Intergenic regions up to 90 bases upstream of identified operons were included in an input set, excluding regions identified in [25] to contain a HrpL promoter element (Hrp box) or that were upstream of an annotated transposase gene. Gibbs sampling using PhyloGibbs [54] was performed to identify promoter-size (approximately 30 nt), conserved motifs, and a motif was consistently found that resembled the known *E. coli *RpoD promoter motif [55]. The aligned sequences were extracted and used to training an HMM for scanning genomic sequences to identify candidate promoters.

For Figure 6 and the associated p-values quoted in the text, the following computations were performed. For every pair of genes within the set of 386 differentially expressed genes shown in Figure 5A, the uncentered correlation (c) of the expression pattern of the genes in that pair was computed, using the same metric with which clustering was performed. A histogram of these correlations forms the blue background distribution labeled "all" in Figure 6(A–D). For each regulatory factor, all possible pairs were formed from the subset of genes predicted to contain an upstream regulatory motif for that factor. The histogram of correlations within each subset is shown in red for each factor. In addition, p-values were computed to estimate the likelihood that the subset of genes predicted to be regulated by a given factor could have been drawn at random from the background distribution of all pair-wise distances. For each factor, 100,000 random subsets of genes of the same size as the predicted subset were drawn from the full set of 386 genes, and all pair-wise correlations were computed for each random subset, from which the mean pair-wise correlation was extracted. The number of random samples with a mean pair-wise correlation that exceeded the mean pair-wise correlation of the predicted gene set for each factor was used to estimate a p-value for that factor. Given the trimodal nature of the background distribution, the mean pair-wise correlation is not a particularly discriminating test statistic, and we therefore expect the quoted p-values to be somewhat conservative, i.e., more discriminating test statistics would be expected to give smaller p-values than those quoted here for the means.

### Microarray data accession

The microarray data from this study is available on the GEO database at  with the accession number GES13500.

## Authors' contributions

PAB contributed to the experimental design, optimized/grew cultures in bioreactors, collected samples, analyzed microarray data, and drafted the manuscript. MJF contributed to experimental design, prepared RNA from culture samples, analyzed microarray data, and edited the manuscript. CRM preformed all clustering analyses and resultant statistical analyses of that data. Additionally CRM was involved in bioinformatics and edited the manuscript. MR performed all ICP-ES experiments and helped to analyze data. DJS contributed to the experimental design, constructed regulatory motifs, was involved in the microarray analysis, subsequent statistical analyses and edited the manuscript. SWC contributed to the experimental design and edited the manuscript.

## Supplementary Material

Additional file 1 **Tables of differentially regulated genes.** This Excel file contains tables of all differentially regulated genes identified with different p value cut-offs. Excel table of genes in the correct order from the pair-wise average linkage with un-centered correlation distance (Figure 5A) and the pair-wise average linkage with absolute un-centered correlation distance (Figure 5B). Click here for file

Additional file 2 **p-0.01_average_uncentered_clustering.cdt.** This CDT file can be used also with the accompanying GTR file to display the gene clustering of differentially regulated genes at GCRMA/FDR p = 0.01 using Java TreeView or similar viewer. Click here for file

Additional file 3 **p-0.01_average_uncentered_clustering.gtr.** This GTR file can be used also with the accompanying CDT file to display the gene clustering of differentially regulated genes at GCRMA/FDR p = 0.01 using Java TreeView or similar viewer. Click here for file

Additional file 4 **p-0.01_average_absolute_uncentered_clustering.cdt.** This CDT file can be used also with the accompanying GTR file to display the gene clustering of differentially regulated genes (without considering the direction of change) at GCRMA/FDR p = 0.01 using Java TreeView or similar viewer. Click here for file

Additional file 5 **p-0.01_average_absolute_uncentered_clustering.gtr.** This GTR file can be used also with the accompanying CDT file to display the gene clustering of differentially regulated genes (without considering the direction of change) at GCRMA/FDR p = 0.01 using Java TreeView or similar viewer. Click here for file

Additional file 6 **Logos of RpoD and Fur motifs.** This png file contains the sequence logos used to scan the DC3000 genome for putative gene targets of these regulators. Click here for file
