# Supplementary figures and images for: Global transcriptional responses of Pseudomonas syringae DC3000 to changes in iron bioavailability in vitro
Source: BMC Microbiol. 2008 Dec 2;8:209. doi: 10.1186/1471-2180-8-209 (PMC2613906; doi:10.1186/1471-2180-8-209)

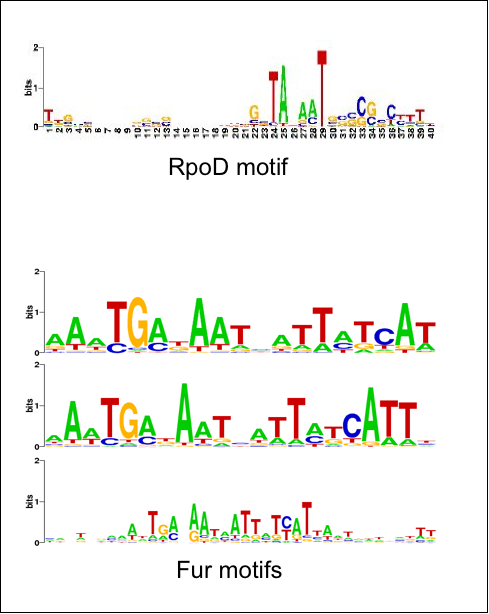

Supplement: Additional file 6 — Logos of RpoD and Fur motifs. This png file contains the sequence logos used to scan the DC3000 genome for putative gene targets of these regulators. [file 1471-2180-8-209-S6.png]
